# Prospective study on the effect of short-term androgen deprivation therapy on PSMA uptake evaluated with ^68^Ga-PSMA-11 PET/MRI in men with treatment-naïve prostate cancer

**DOI:** 10.1007/s00259-019-04635-7

**Published:** 2019-12-26

**Authors:** Otto Ettala, Simona Malaspina, Terhi Tuokkola, Pauliina Luoto, Eliisa Löyttyniemi, Peter J. Boström, Jukka Kemppainen

**Affiliations:** 1 Department of Urology, University of Turku and Turku University Hospital, Turku, Finland; 2 Department of Clinical Physiology and Nuclear Medicine, University of Turku and Turku University Hospital, Turku, Finland; 3 Turku PET Centre, University of Turku and Turku University Hospital, Turku, Finland; 4 Department of Biostatistics, University of Turku, Turku, Finland

**Keywords:** Prostate cancer, PSMA, PET, androgen deprivation therapy, ADT

## Abstract

**Purpose:**

Based on in vitro studies, it is known that androgen deprivation therapy (ADT) increases prostate-specific membrane antigen (PSMA) expression. Therefore, we hypothesised that ADT improves the performance of PSMA-PET imaging in primary staging of prostate cancer. The purpose of the study was to demonstrate the time course effect of ADT on PSMA uptake in different types of metastatic lesions evaluated with ^68^Ga-PSMA-11 PET/MRI.

**Methods:**

Nine men with treatment-naïve prostate cancer were enrolled to a prospective, registered (NCT03313726) clinical trial. A ^68^Ga-PSMA-11 PET/MRI was performed once before and 3 times post-ADT (degarelix, Firmagon). Change of maximum standardised uptake values (SUVmax) in prostate, lymph nodes, bone metastases, and physiologically PSMA-avid organs were evaluated in a time frame of 1–8 weeks.

**Results:**

All patients reached castration levels within 10 days, and 50% decrease in prostate-specific antigen (PSA) concentration was observed 14 days post-ADT. A heterogeneous increase in PSMA uptake was observed 3 to 4 weeks post-ADT. This phenomenon was definitively more evident in bone metastases: 13 (57%) of the metastasis, with a mean (range) SUVmax increase of 77% (8–238%). In one patient, already having bone metastases at baseline, three new bone metastases were observed post-ADT. Of lesions with reduced SUVmax, none disappeared.

**Conclusions:**

Both in patient and region level, increase in PSMA uptake post-ADT is heterogenous and is seen most evidently in bone metastases. Preliminary results on a small cohort of patients suggest the clinical impact of ADT on improving the performance of ^68^Ga-PSMA PET in staging seems to be minor. However, the optimal imaging time point might be 3 to 4 weeks post-ADT. Since none of the metastases with decreasing SUVmax disappeared, it seems that short-term usage of ADT does not interfere with the interpretation of ^68^Ga-PSMA PET.

**Trial registration:**

NCT03313726, registered 18 October 2017; EUDRA-CT, 2017-002345-29.

**Electronic supplementary material:**

The online version of this article (10.1007/s00259-019-04635-7) contains supplementary material, which is available to authorized users.

## Introduction

Currently, small-molecule imaging with gallium- or fluoride-labelled prostate-specific membrane antigen (^68^Ga/^18^F-PSMA) has been rapidly taken into clinical use in many European countries [1–3], although its utility in primary staging of prostate cancer still needs further validation [4–6]. At present, according to the majority of published data, the main indication of ^68^Ga-PSMA PET imaging is re-staging in presence of biochemical recurrence, especially at low prostate-specific antigen (PSA) values [7]. However, in recent years, there has been also a growing evidence on the promising role of ^68^Ga-PSMA PET imaging in nodal and distant staging in patients with high-risk disease [8].

Based on in vitro studies and animal models, it is known that administration of androgen deprivation therapy (ADT) increases PSMA expression [9, 10]. Although this notion was primarily published more than 20 years ago [9], first clinical case report was published only 2 years ago. Hope et al. demonstrated a 7-fold increase in maximum standardised uptake value (SUVmax) of PSMA uptake after the initiation of ADT [10]. However, in two recent series, the effect of ADT on PSMA-PET findings was deemed as heterogeneous [11, 12]. However, in both of these series, all the metastases were analysed as whole and no region-based analysis was performed. In addition, no data is present if increase is also seen in physiologically avid organs. According to previous published data, we hypothesised that ADT might improve the performance of PSMA-PET imaging in primary staging of prostate cancer. In addition, the hypothesis was that different regions of prostate cancer (primary tumour in prostate and metastases in lymph nodes, bone, viscera) and physiologically avid organs as well (salivary glands, kidneys, liver, and spleen) respond differently to ADT.

The purpose of the current study is to demonstrate in patient and region-based analysis of the time course effect of ADT on PSMA uptake observed in repeated ^68^Ga-PSMA-11 PET/MRI scans in men with newly diagnosed, treatment-naïve prostate cancer patients.

## Materials and methods

### Study population and design

In this prospective, registered (NCT03313726) clinical trial, men with newly diagnosed, treatment-naïve, high-risk prostate cancer with high risk for metastases were enrolled. In seven patients, 12-core TRUS-guided biopsies were performed, while in two patients suffering from urinary retention, the diagnosis was made from specimens obtained from transurethral resection of the prostate (TURP). The inclusion criteria were (1) histologically confirmed adenocarcinoma of the prostate and (2) no previous surgical, radiation, or endocrine treatment of the prostate cancer. Exclusion criteria were (1) presence of uncontrolled serious infection and (2) contraindications for MRI imaging. Also, since 5-alpha-reductase inhibitors, namely finasterid and dutasterid, affect the steroid pathway and possibly the PSMA uptake, men with prior usage of 5-alpha-reductase inhibitor medication in the past 12 months were excluded.

A ^68^Ga-PSMA-11 PET/MRI was performed before and 3 times after the subcutaneous administration of ADT (degarelix). The post-ADT PSMA-PET/MRI scans were performed at a mean (range) of 1.5 (0.8–2.5) weeks, 2.9 (1.9–4.5) weeks, and 6.2 (3.5–8.7), respectively.

PSA and testosterone blood samples were collected before every scan. After the study, all patients were treated based on current institutional guidelines.

### Ethical issues

The study was conducted in compliance with the current revision of Declaration of Helsinki guiding physicians and medical research involving human subjects (64th World Medical Association General Assembly, Fortaleza, Brazil, 2013). All patients signed a written informed consent, and the study received the approval of Finnish Medicines Agency (FIMEA; EUDRA-CT, 2017-002345-29) and the Ethical Committee of the Hospital District of Southwest Finland.

### PSMA-PET/MRI imaging protocol

PSMA-PET/MRI scans were performed using a sequential Philips Ingenuity time-of-flight (TF) PET/MR scanner (Philips Healthcare, Cleveland, OH). All patient received an intravenous injection of ^68^Ga-PSMA-11 (mean ± SD administered activity, 153 ± 10 MBq). After 20 min from radiotracer injection, MRI scanning protocol started with T2-weighted turbo-spin-eco sequences in transaxial, coronal, and sagittal direction, and diffusion-weighted sequence using a dedicated external coil for the lower abdomen (SENSE-TORSOXL). Subsequently, whole-body T2-weighted and an MRI-based attenuation correction sequence were obtained. PET whole-body acquisition from the orbital region to the mid-thighs (approximately 10 table positions, 4 min/table) started 64 ± 3 min (mean ± SD) from radiotracer injection. PET imaging reconstructions were performed using the default reconstruction algorithm “Blob-OS-TF”, a 3D ordered subset iterative TOF reconstruction technique. The reconstruction used 3 iterations and 33 subsets in 144 × 144 matrix with an isotropic voxel size of 4 mm. All reconstructions included the necessary corrections for image quantification: attenuation, random, scatter, dead-time, decay, and detector normalisation.

### Image analysis

Image analysis was performed using an AW 4.5 workstation by General Electrics (GE) Healthcare. Two experienced nuclear medicine physicians analysed the images blinded for the results of the other reader but unblinded for other imaging modalities and clinical data available. In case of equivocal findings, a consensus between the two readers was reached in a multidisciplinary board meeting.

Volumes of interest (VOIs) were drawn on PSMA positive prostate lesions, lymph nodes, and bone metastases. Similar VOIs were drawn in salivary glands (parotid, submandibular, and sublingual), liver, spleen, and kidneys (avoiding the renal pelvis). PSMA uptake was measured using the standardised uptake value maximum (SUVmax) values and ΔSUVmax at different time points was calculated compared to the pre-ADT scan.

Findings on PSMA-PET scans were interpreted according to the current suggested procedure guideline on ^68^Ga-PSMA PET imaging, taking into consideration normal biodistribution of the tracer and possible pitfalls [13]. PSMA-positivity was defined as a focal tracer uptake higher than adjacent background on prostate, suspicious bone, and lymph node lesions, without using a strict cut-off value of SUVmax to indicate or confirm malignancy. Moreover, any anatomical or functional correspondence on MRI imaging, such as altered signal on T2w and/or diffusion restriction on DWI or LN diameter and morphology, was also used in guiding the interpretation.

### Statistical methods

In order to differentiate the effect of ADT in region level in Figs. 2 and 3, and Table 3, lesions were divided and analysed in two groups: “decrease”, lesions in which, when comparing to baseline, change in SUVmax was constantly negative in every time point; “increase”, all other lesions. In Table 3, the two groups were further analysed by evaluating the maximum increase and maximum decrease in SUVmax from each lesion by selecting a time point at which the highest or the lowest SUVmax occurred. At this specific time point, the change in SUVmax was described as mean proportion (range) and the time point as mean weeks (range). To evaluate inter-reader agreement, Cohen’s Kappa (95% CI) was calculated.

## Results

Nine patients were included in the study. Patients’ characteristics are shown in Table 1. At baseline, five had metastatic prostate cancer (other than regional lymph nodes and/or bone), while four patients presented with local or locally advanced disease. Visceral metastases were not present in any of the patients. All patients reached castration levels (serum testosterone < 1.7 nmol/L) within a period of 10 days after the initiation of ADT. Eight patients completed all the four PET/MRI scans, while one patient performed only three scans due to study withdrawal.

**Table 1:** Patient characteristics. *PSA*, prostate-specific antigen; *S-Testo*, serum testosterone; *cT*, clincal T stage; *cN*, clinical N stage; *cM*, clinical M stage according to PSMA-PET

	Age (years)	PSA (μg/l)	S-Testo (nmol/L)	Gleason score	cT	cN	cM
Patient 1	64	21	26	4 + 5	2c	1	1
Patient 2	69	25	13	4 + 5	3a	0	0
Patient 3	69	7	19	5 + 5	3a	0	0
Patient 4	77	7	7	4 + 5	1b	1	1
Patient 5	66	280	10	4 + 5	2a	0	1
Patient 6	71	52	9	5 + 4	3a	1	1
Patient 7	78	54	23	5 + 4	4	1	0
Patient 8	70	26	18	5 + 4	1b	1	1
Patient 9	70	9	12	5 + 3	2a	0	0

**Fig. 1:**
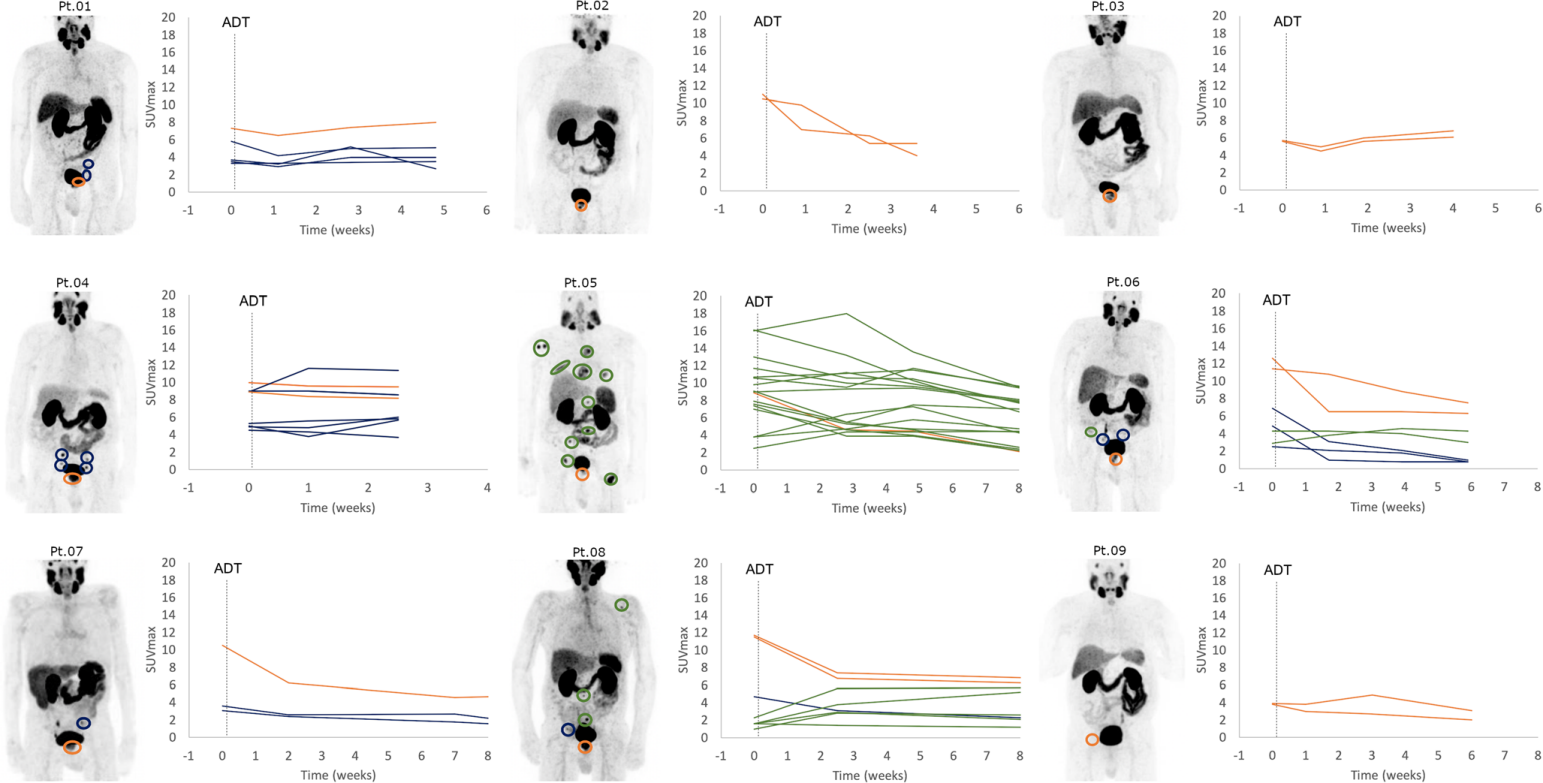
Patient-based changes in SUVmax after the administration of ADT (degarelix). Orange line, prostate lesions; blue line, lymph node metastases; green lines, bone metastases; dotted vertical line, initiation of androgen deprivation therapy (ADT)

Patient-based observations are depicted in Fig. 1. In two patients (patients no. 2 and no. 7), no increase in SUVmax was observed, whereas in 7 (78%) patients, a heterogeneous change in PSMA uptake occurred. In patient no. 8, who had two bone metastases already at baseline, three new bone metastases were observed post-ADT.

**Fig. 2:**
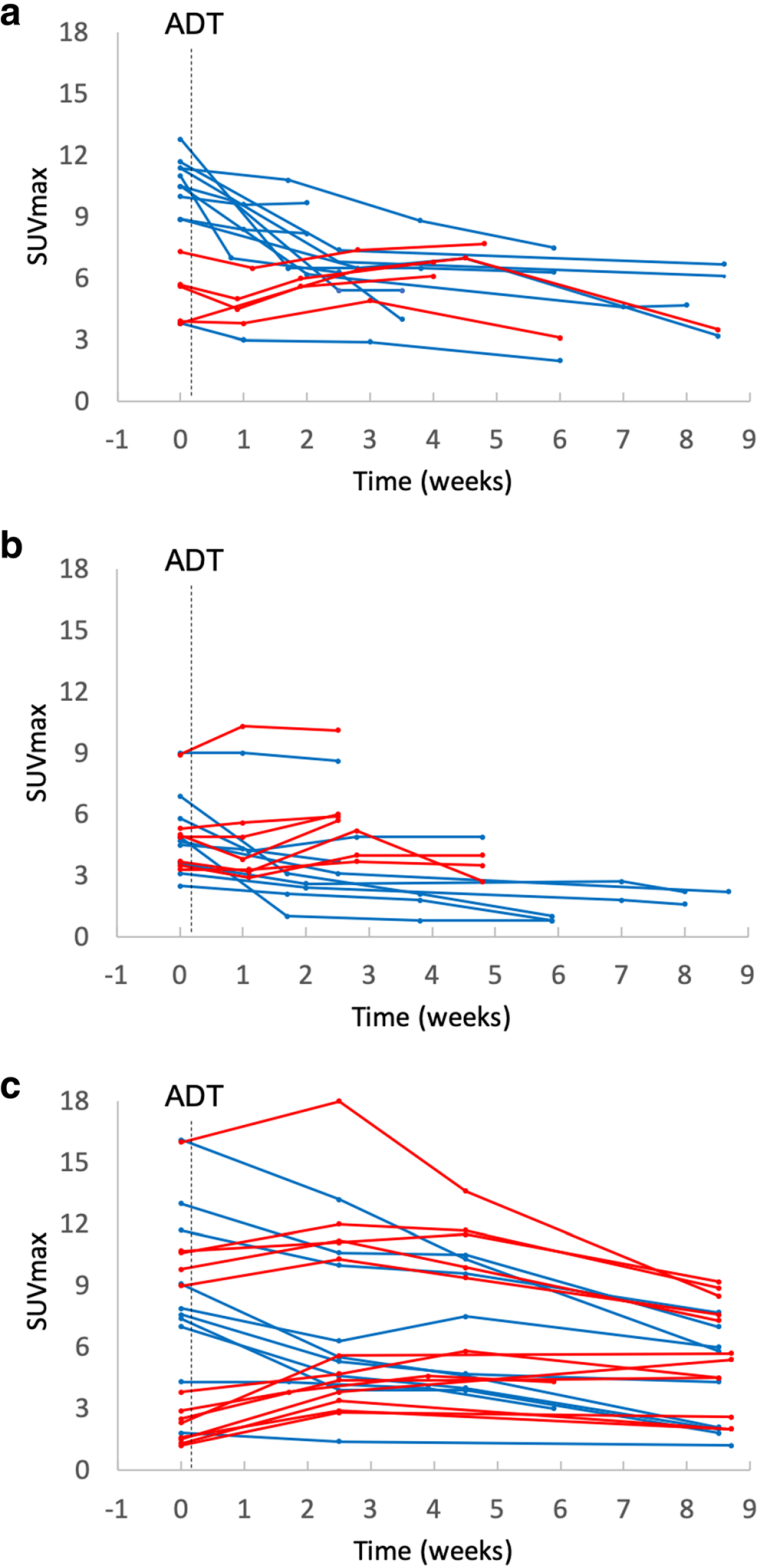
Lesion-based SUVmax trend in prostate lesions (**a**), lymph nodes (**b**), and bone metastases (**c**). Blue lines, lesions with decreasing SUVmax trend compared with baseline; red lines, lesions with increasing SUVmax trend compared with baseline; dotted vertical line, initiation of androgen deprivation therapy (ADT)

**Fig. 3:**
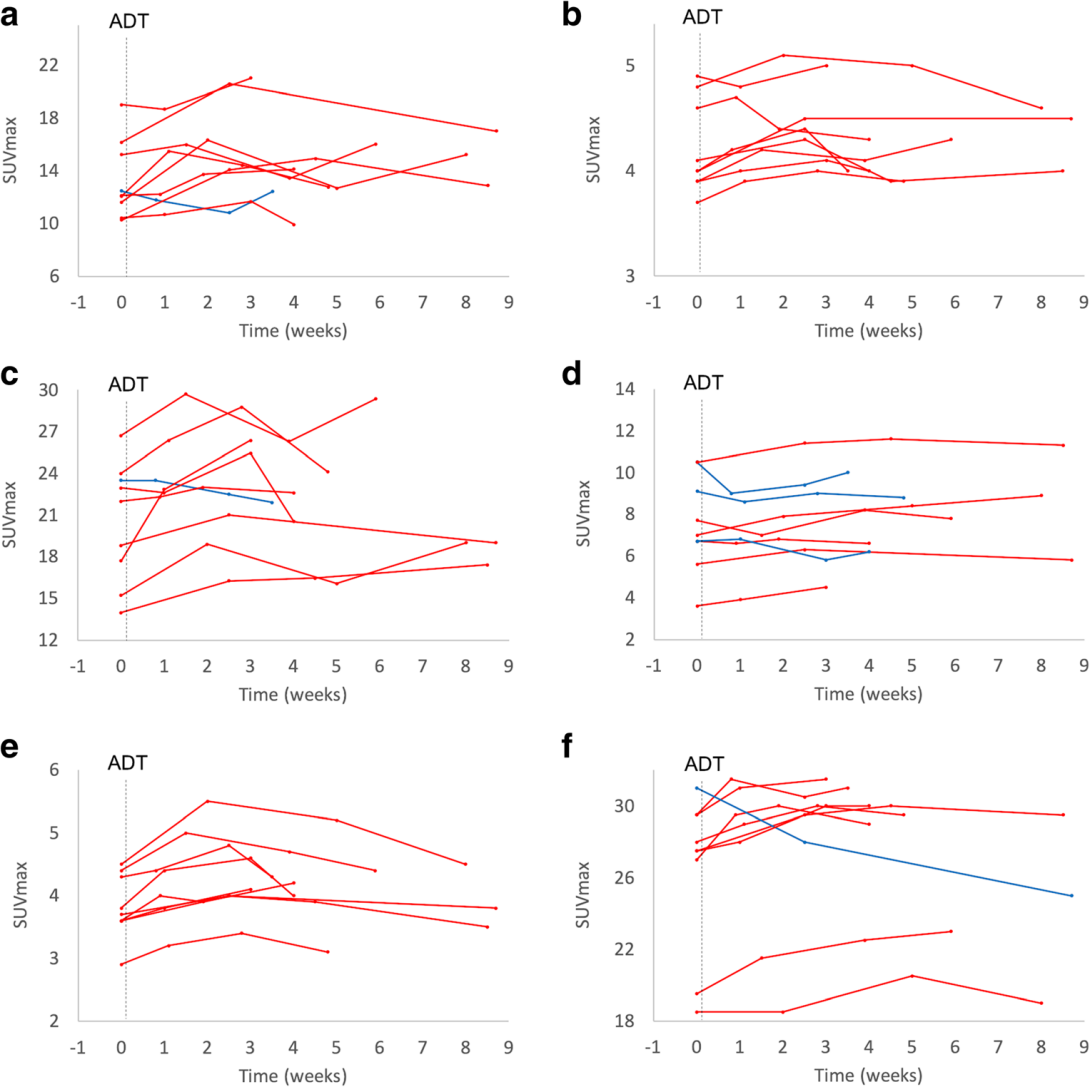
SUVmax trend in parotid glands (**a**), liver (**b**), submandibular glands (**c**), spleen (**d**), sublingual glands (**e**), and kidneys (**f**) after initiation of ADT (degarelix): blue lines, lesions with decreasing SUVmax; red lines, lesions with increasing SUVmax trend compared with baseline; dotted vertical line, initiation of androgen deprivation therapy (ADT)

A region-based analysis of the primary tumour in prostate, metastatic lesions, and the uptake pattern in physiologically avid organs is depicted in Figs. 2 and 3, and in Table 2. Also, the specific locations of the metastases are presented in Table 3. All the lesions in baseline and in follow-up, except for a discordance in two parailiac lymph node metastases, were detected by the two readers, Cohen’s Kappa 0.89 (95% CI, 0.79–0.99). After a consensus reading, in total, 16 prostate, 16 lymph node, and 23 bone lesions were detected and analysed.

**Table 2:** Location of metastases

Lymph nodes	*n* (%)
Parailiacal	13 (33)
Mesorectal	1 (3)
Para-aortic	2 (5)
Bone	*n* (%)
Humerus	1 (3)
Sternum	2 (5)
Scapula	1 (3)
Ribs	2 (5)
Cervical vertebra	1 (3)
Thoracic vertebra	3 (7)
Lumbar vertebra	4 (10)
Iliac bone	7 (17)
Sacrum	1 (3)
Femur	1 (3)

**Table 3:** Maximum increase and maximum decrease of maximum standardised uptake values. (SUVmax) in prostate, lymph node, bone lesions, and normal PSMA-avid organs. SUVmax is presented in proportional change and the time point at which the maximum SUVmax occurred is presented in weeks. Paired organs are analysed as a mean of right and left

	Maximum increase			Maximum decrease		
Lesion/normal organ	*n*	SUVmax (%); mean (range)	Time point (weeks); mean (range)	*n*	SUVmax (%); mean (range)	Time point (weeks); mean (range)
Primary tumour (prostate)	5	29 (6–84)	4.3 (3.0–4.8)	11	− 46 (− 64–(− 4))	5.8 (1.0–8.6)
Lymph node metastases	7	19 (11–41)	2.7 (1.0–4.8)	9	− 48 (− 86–(− 4))	5.2 (1.1–8.7)
Bone lesions	13	76 (8–238)	4.3 (2.5–8.7)	10	− 50 (− 77–(− 20))	7.7 (2.5–8.7)
Parotid glands	8	23 (5–45)	3.3 (1.1–5.9)	1	− 8	3.5
Submandibular glands	8	22 (5–49)	4.1 (1.7–8.5)	1	− 14	2.5
Sublingual glands	9	15 (8–22)	2.7 (1.7–4.0)	–	–	–
Liver	9	7 (2–13)	2.8 (0.9–5.9)	–	–	–
Spleen	6	16 (2–36)	4.0 (1.9–8.0)	3	− 19 (− 6–(− 14))	1.6 (0.8–3.0)
Kidneys	8	10 (7–18)	3.6 (0.8.7.0)	1	− 19	8.7

There was a marked increase in SUVmax (maximum increase in SUVmax of 76%) in more than half of the bone metastasis and a less pronounced and not so frequent increase in lesions in prostate (29%), and lymph nodes (19%). The increase was observed within the first 3 to 4 weeks post-ADT. In all lesions, which were considered “decrease”, the maximum SUVmax decrease was 50% or less. Despite the decrease, none of the lesions disappeared during the follow-up.

**Fig. 4:**
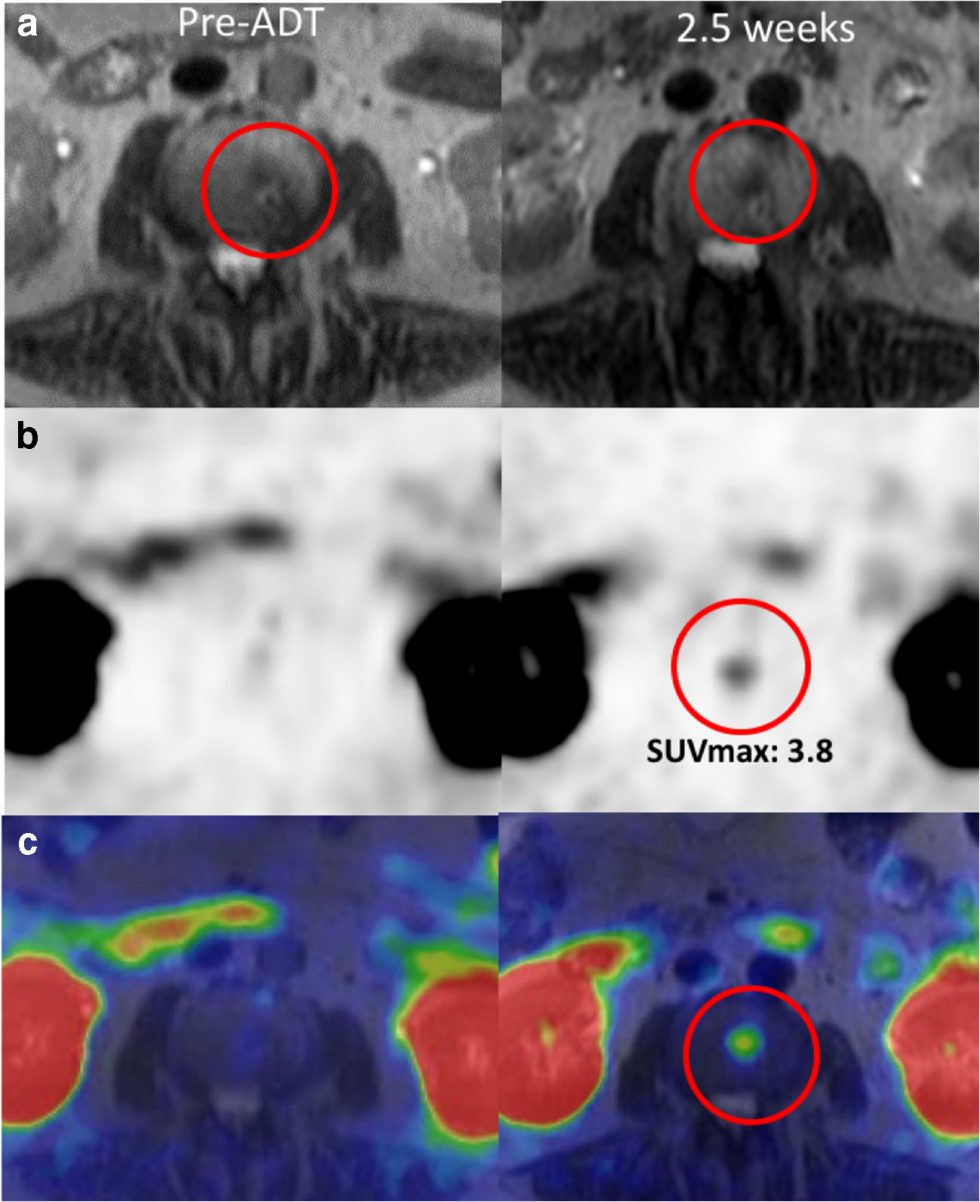
T2W-MRI (**a**), PET (**b**), and fused PET/MRI (**c**) images of patient no. 8 at baseline and 2.5 weeks after ADT (degarelix). A new bone metastasis in L2 vertebra was detected. The lesion was already visible at baseline T2W-MRI images but PSMA uptake occurred 2.5 weeks after the initiation of ADT

The most pronounced increase in physiologically avid organs was observed in parotid glands (23%), and submandibular glands (22%). In other physiologically avid organs, the increase was less than 20%. The decrease was seen very seldom or not at all, and the mean decrease was less than 20% (Fig. 4).

## Discussion

In this prospective registered study, a heterogeneous increase of PSMA uptake was observed after ADT in treatment-naïve prostate cancer patients in repeated ^68^Ga-PSMA PET/MRI scans. The most evident increase in SUVmax was observed 3 to 4 weeks post-ADT especially in bone metastasis. In one patient, with bone metastases already at baseline, three new bone metastases, which were already visible anatomically on MRI images, were observed. Of those lesions with decreasing SUVmax, none disappeared.

It has already been demonstrated that in both androgen-sensitive and androgen-resistant human prostate adenocarcinoma cells (LNCaP), the expression of PSMA is upregulated post-ADT and downregulated in the presence of testosterone or DHT [14–16]. Although a case report published by Hope et al. corroborated these preclinical studies, demonstrating a 7-fold increase in PSMA SUVmax values after the initiation of ADT, our results are in line with the two very recently published studies [10–12]. Aggarwal et al. performed a study in eight patients, scanned with ^68^Ga-PSMA-11 PET before and after the initiation of ADT within a variable period of 2–4 weeks. The study demonstrated a heterogeneous increase in SUVmax, in 68% and in 41% of lesions in castration-sensitive men (*n* = 4) and in castration-resistant men (n = 4), respectively. The other study, conducted by Emmet et al., studied also both castration-sensitive (*n* = 8) and castration-resistant men (*n* = 7). Although they showed that the increase was more evidently seen in castration-resistant men, in the light of our data and the study by Aggarwal, it seems that the increase occurs also in castration-sensitive men. In fact, in the study by Emmet et al., of those eight men with castration-sensitive disease, half of them had Gleason score 7 prostate cancer. It might well be that the increase is more evidently seen in poorly differentiated tumours. Also, our study clearly demonstrates the biological difference between testosterone flare and PSMA flare. Since degarelix directly inhibits the action of GnRH, no PSA flare occurs, and serum testosterone decreases rapidly. Despite the rapid decrease in testosterone levels, the increase in PSMA uptake was still observed.

ADT is the gold standard treatment in patients with metastatic prostate cancer. The effect of continuous long-term ADT on reducing the visibility of castration-sensitive prostate cancer lesions on PSMA-PET has already been investigated; however, it is still uncertain if initiation of ADT could interfere with the staging results [17]. According to our preliminary findings, as none of the lesions disappeared during the observational period, ongoing short-term ADT does not represent a contraindication on performing a staging PSMA-PET. Although ADT did not significantly increase PSMA-PET staging performance as only in one patient new metastases were found, PSMA activity in the majority metastatic lesion reached the highest uptake 3 to 4 weeks post-ADT. Therefore, this time window might improve the detection rate, providing better lesion to background ratio. This aspect might be interesting especially in an oligometastatic disease, where bone metastases detected could then be selectively treated with radiotherapy. However, further studies are warranted to investigate the possible clinical impact of the phenomenon.

Since this is the first study where changes in PSMA SUVmax values were analysed on a region-based analysis, the fact that we observed the increase most evidently in bone metastasis is also interesting. This notion is supported also by two case reports demonstrating a similar increase of PSMA uptake in bone metastasis [18, 19]. Although the biology of the phenomenon is not known, authors hypothesised that increase of PSMA uptake in bone might be caused by osteoblastic turn over or bone reparation processes. However, most probably, this is not the case, since we observed the increase also in lymph nodes, in primary tumours, and in some of the physiologically avid organs suggesting a more general rather than organ-specific mechanism. This is, in fact, the first study to report that changes in PSMA-PET findings post-ADT are not restricted only to tumour tissue.

One might also question whether this flare phenomenon is dependent on the different mechanisms of action of ADT treatments. However, our results with GnRH antagonist therapy are similar with previous studies, in which LhRH agonist, antiandrogens, or new androgen signalling pathway modulators were administered [10–12, 16]. Given these facts, it seems rather evident that PSMA is connected to the androgen pathway [10–12, 16]. More recently, also mTOR pathway with mTOR inhibitor, rapamycin has shown to be linked to increase in PSMA uptake [16, 20]. Taken this all together, one might hypothesise that the observed effects of ADT on PSMA uptake in lesion level depict the androgen sensitivity of the specific lesions and also potentially the heterogeneity in aggressiveness of the lesions. Therefore, could it be possible to select those lesions that are prone to progress or are insensitive to ADT and selectively treat only those lesions? To understand this phenomenon, further studies with larger number of patients and longer follow-up are warranted.

Moreover, understanding what lies behind this phenomenon might raise a great interest from a theragnostic perspective. A preclinical study on a mouse model of castration-resistant prostate cancer (CRPC) demonstrated that pre-treatment with enzalutamide for 21 days followed by 177Lu-PSMA radioligand therapy (RLT) resulted in a significantly enhanced RLT-induced DNA-damage. However, pre-treatment with androgen receptor blockade did not show any additive effect on tumour growth reduction, suggesting that ADT might not necessarily guarantee an increased efficacy of RLT [21]. Nevertheless, considering the heterogeneity of PSMA expression and the lack of clinical studies about this specific theragnostic aspect, the possible synergistic effect of ADT + RLT still needs to be defined.

Moreover, although ^177^Lu-PSMA radionuclide therapy is at the present moment used in castration-resistant prostate cancer patients, there are ongoing clinical trials to study its use also in castration-sensitive patients [22]. If proven to be effective in castration-sensitive men, according to our data, it would be reasonable to hypothesise that the timing between administration of ADT and of ^177^Lu-PSMA radionuclide therapy is crucial. ADT could increase the tumour targeting and therefore increase the efficacy of ^177^Lu-PSMA treatment during the time window of maximum PSMA uptake.

Also, our finding of increased PSMA uptake in normal salivary glands post-ADT needs to be taken into account as a possible increased therapy-related risk factor for significant xerostomia in patient candidates for ^177^Lu-PSMA therapy.

The study has some limitations. First, the cohort is small and underpowered to infer firm conclusions. In addition, there is variability in time intervals of the scans between the different patients. Therefore, the results should be considered preliminary and the study as a proof of concept. However, all the patients were thoroughly examined, and eight of the nine patients completed the study by undergoing four sequential PSMA-PET/MRI scans. In addition, the strength of the study is its truly prospective and registered nature. Second, the cohort is heterogenous. However, this can also be seen as an advantage since with this cohort, we are able to demonstrate that, although the increase is minor, PSMA flare is not merely seen in metastatic patients.

## Conclusions

Both in patient and region level, a heterogeneous increase of PSMA uptake was observed post-ADT, observed most evidently in bone metastases. The highest response on PSMA uptake was observed 3 to 4 weeks after ADT. Although the impact of ADT on ^68^Ga-PSMA PET staging performance was minor in this small patient cohort, more research is needed to investigate whether ADT could significantly improve detection rate and have clinical impact in patients with oligometastatic disease. Moreover, results were encouraging that short-term usage of ADT does not seem to represent a contraindication to perform 68Ga-PSMA PET for staging purpose, since none of the lesions disappeared.

## Electronic supplementary material

Figure S1 Serum testosterone decline after administration of androgen deprivation therapy (ADT). Grey line, single patient; blue line, mean trend. Dotted line, initiation of androgen deprivation therapy (ADT) (PNG 132 kb)

High Resolution Image (TIF 391 kb)

Figure S1 Plasma PSA decline after administration of androgen deprivation therapy (ADT). Grey line, single patient; blue line, mean trend. Dotted line, initiation of androgen deprivation therapy (ADT) (PNG 130 kb)

High Resolution Image (TIF 384 kb)
