# Supplementary figures and images for: Prospective study on the effect of short-term androgen deprivation therapy on PSMA uptake evaluated with 68Ga-PSMA-11 PET/MRI in men with treatment-naïve prostate cancer
Source: Eur J Nucl Med Mol Imaging. 2019 Dec 26;47(3):665–73. doi: 10.1007/s00259-019-04635-7 (PMC7081750; doi:10.1007/s00259-019-04635-7)

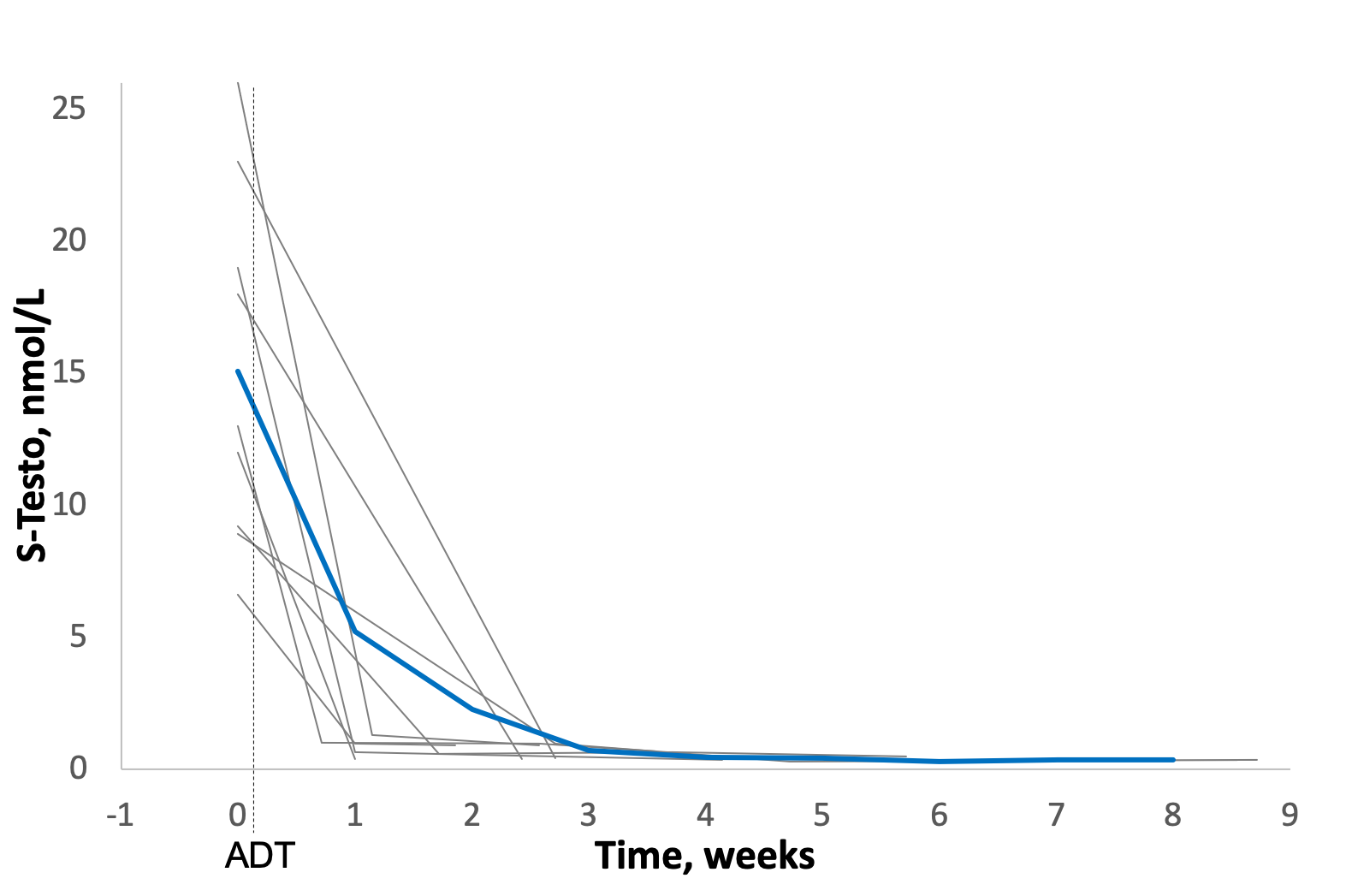

Supplement: Supplementary file 1 — Serum testosterone decline after administration of androgen deprivation therapy (ADT). Grey line, single patient; blue line, mean trend. Dotted line, initiation of androgen deprivation therapy (ADT) (PNG 132 kb) [file 259_2019_4635_MOESM1_ESM.png]

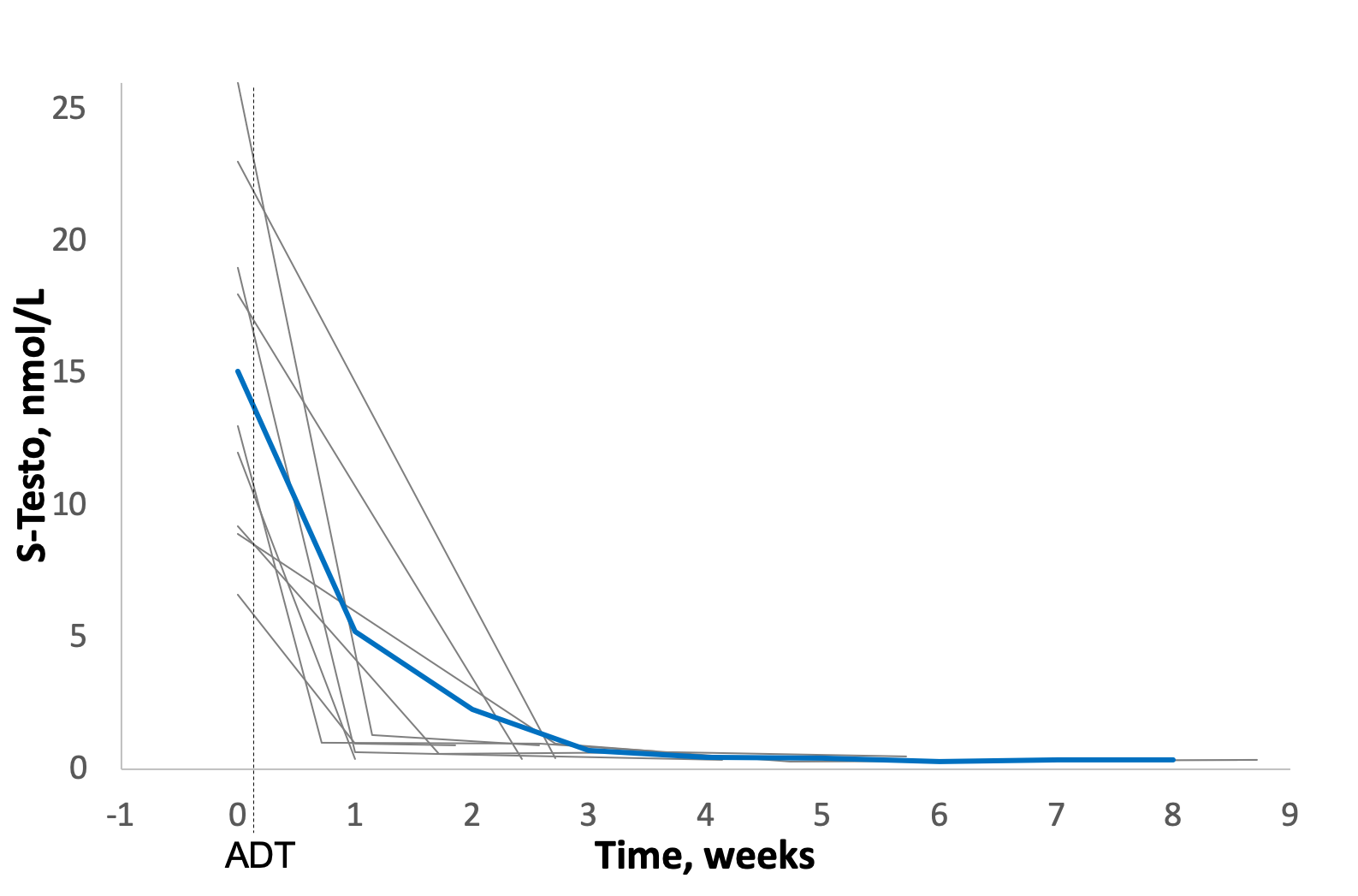

Supplement: Supplementary file 2 — High Resolution Image (TIF 391 kb) [file 259_2019_4635_MOESM5_ESM.tif]

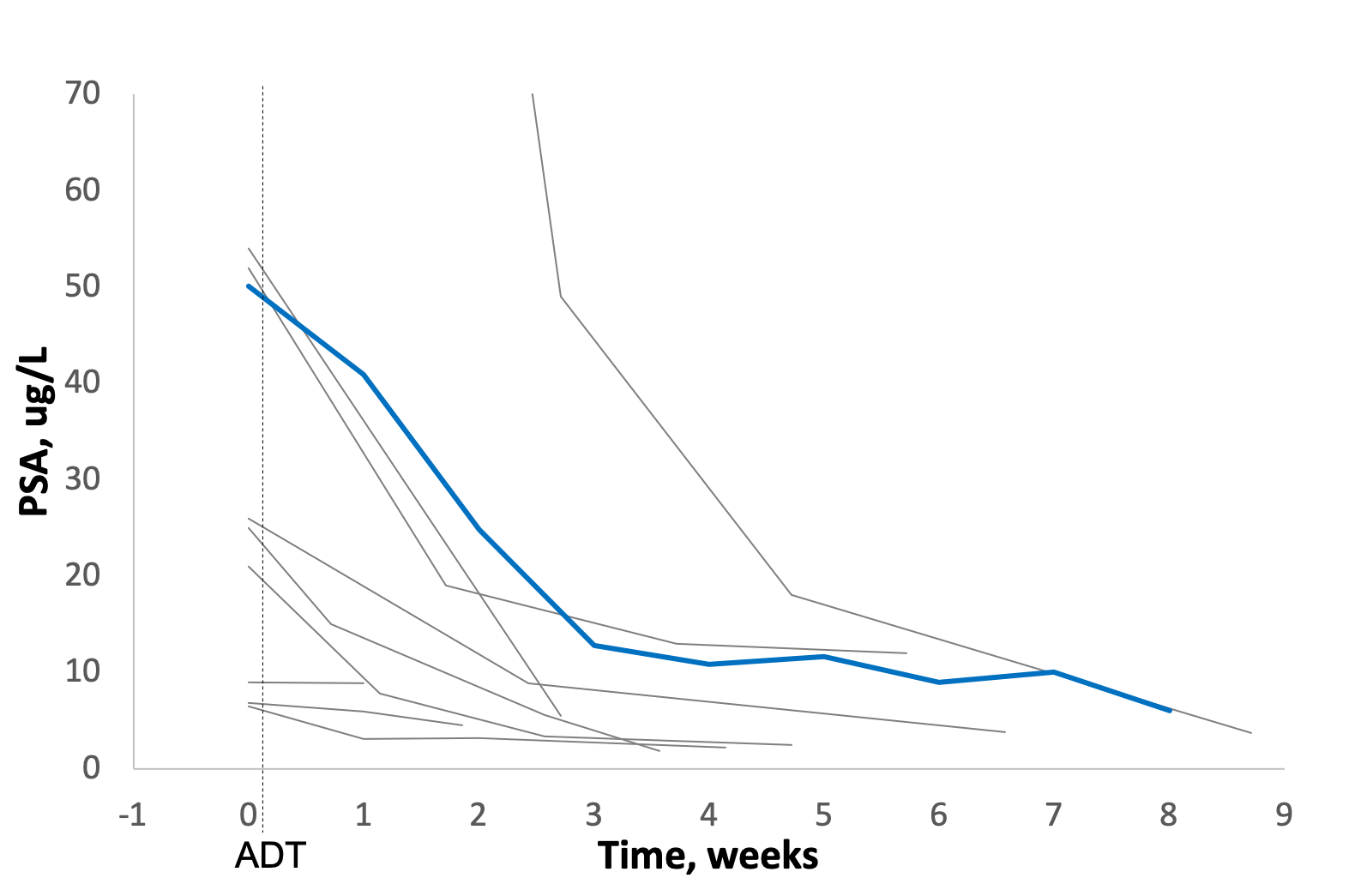

Supplement: Supplementary file 3 — Plasma PSA decline after administration of androgen deprivation therapy (ADT). Grey line, single patient; blue line, mean trend. Dotted line, initiation of androgen deprivation therapy (ADT) (PNG 130 kb) [file 259_2019_4635_MOESM2_ESM.png]

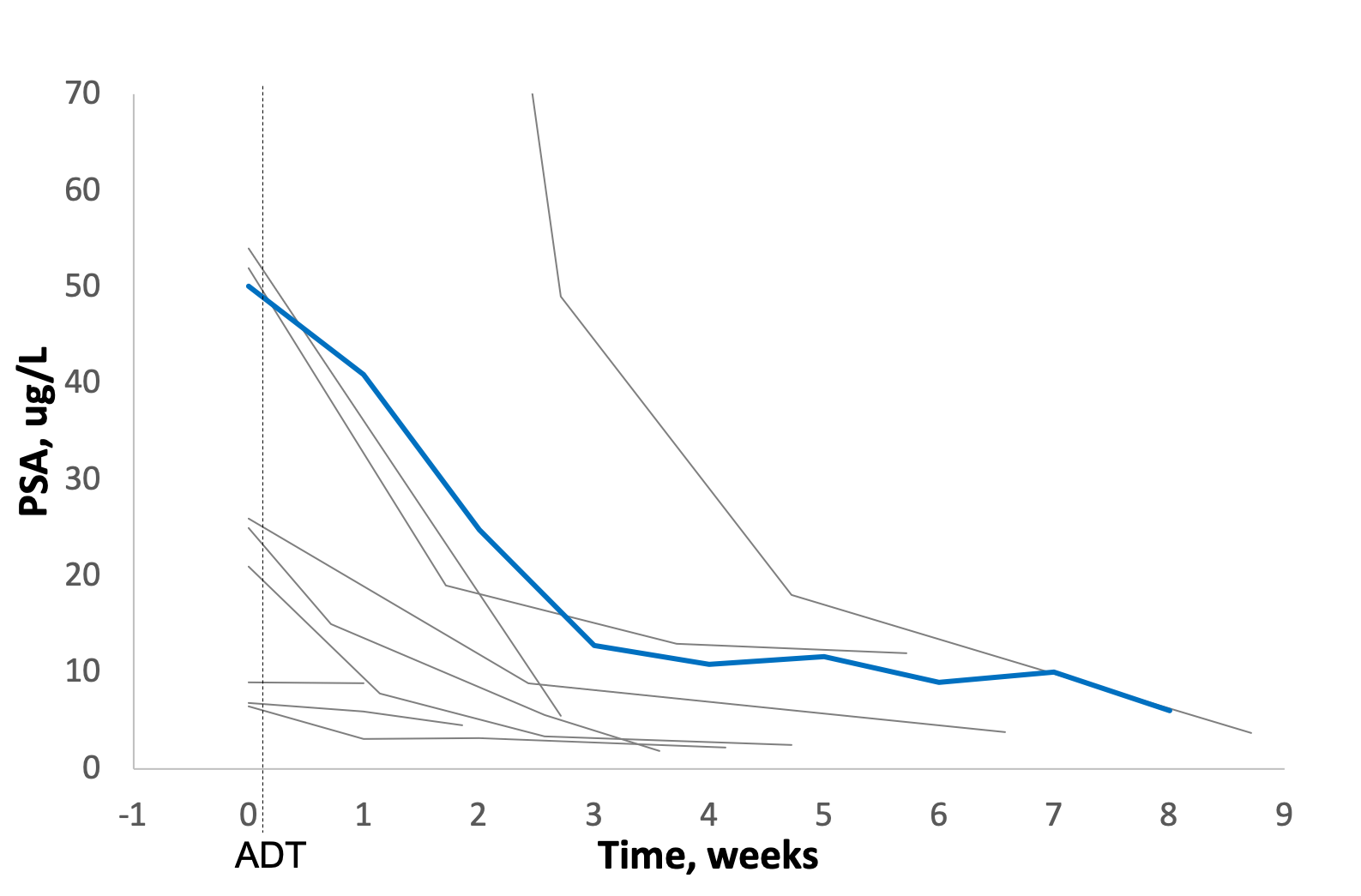

Supplement: Supplementary file 4 — High Resolution Image (TIF 384 kb) [file 259_2019_4635_MOESM6_ESM.tif]
